# Transcriptome based individualized therapy of refractory pediatric sarcomas: feasibility, tolerability and efficacy

**DOI:** 10.18632/oncotarget.25087

**Published:** 2018-04-17

**Authors:** Bushra Weidenbusch, Günther H.S. Richter, Marie Sophie Kesper, Monika Guggemoos, Katja Gall, Carolin Prexler, Ilya Kazantsev, Alexandra Sipol, Lars Lindner, Michaela Nathrath, Olaf Witt, Katja Specht, Frigga Beitinger, Carolin Knebel, Stuart Hosie, Rüdiger von Eisenhardt-Rothe, Wilko Weichert, Irene Teichert-von Luettichau, Stefan Burdach

**Affiliations:** ^1^ Department of Pediatrics and Children’s Cancer Research Center, Kinderklinik München Schwabing, Klinikum rechts der Isar, Fakultät für Medizin, Technische Universität München, Munich, Germany; ^2^ Department of Pharmacology, Städtisches Klinikum München GmbH, Munich, Germany; ^3^ Department of Hematology/Oncology, Munich University Hospital, Ludwig-Maximilians-Universität München, Munich, Germany; ^4^ RM Gorbacheva Scientific Research Institute of Pediatric Hematology and Transplantation, Pavlov First Saint Petersburg State Medical University, Saint Petersburg, Russia; ^5^ Department of Pediatric Surgery, Städtisches Klinikum München GmbH, Munich, Germany; ^6^ Department of Pediatric Hematology and Oncology, Klinikum Kassel, Germany; ^7^ Department of Pediatric Oncology, Hematology and Immunology, University Hospital Heidelberg, Heidelberg, Germany; ^8^ Institute of Pathology, Technische Universität München, Munich, Germany; ^9^ Department of Pathology, Städtisches Klinikum München GmbH, Munich, Germany; ^10^ Department of Orthopedic Surgery, Klinikum rechts der Isar, Fakultät für Medizin, Technische Universität München, Munich, Germany; ^11^ CCC München - Comprehensive Cancer Center; and DKTK German Cancer Consortium Munich, Munich, Germany

**Keywords:** targeted therapy, pediatric cancer, sarcoma, adolescents and young adults, expression profiling

## Abstract

Survival rates of pediatric sarcoma patients stagnated during the last two decades, especially in adolescents and young adults (AYAs). Targeted therapies offer new options in refractory cases. Gene expression profiling provides a robust method to characterize the transcriptome of each patient’s tumor and guide the choice of therapy.

Twenty patients with refractory pediatric sarcomas (age 8-35 years) were assessed with array profiling: ten had Ewing sarcoma, five osteosarcoma, and five soft tissue sarcoma. Overexpressed genes and deregulated pathways were identified as actionable targets and an individualized combination of targeted therapies was recommended. Disease status, survival, adverse events (AEs), and quality of life (QOL) were assessed in patients receiving targeted therapy (TT) and compared to patients without targeted therapy (non TT).

Actionable targets were identified in all analyzed biopsies. Targeted therapy was administered in nine patients, while eleven received no targeted therapy. No significant difference in risk factors between these two groups was detected. Overall survival (OS) and progression free survival (PFS) were significantly higher in the TT group (OS: P=0.0014, PFS: P=0.0011). Median OS was 8.83 versus 4.93 months and median PFS was 6.17 versus 1.6 months in TT versus non TT group, respectively. QOL did not differ at baseline as well as at four week intervals between the two groups. TT patients had less grade 1 AEs (P=0.009). The frequency of grade 2-4 AEs did not differ.

Overall, expression based targeted therapy is a feasible and likely beneficial approach in patients with refractory pediatric sarcomas that warrants further study.

## INTRODUCTION

Despite advances in the treatment of pediatric bone and soft tissue sarcomas (STS) survival rates have stagnated especially in refractory cases. Treatment options are still limited to surgery, radiotherapy and conventional cytotoxic chemotherapy regimens. In the era of genomic medicine a wide range of new therapeutic agents has been developed that target specific genomic alterations and pathways key to cancer cell survival and proliferation, a concept which is termed “targeted therapy” [1]. In many adult solid tumor entities these novel drugs are highly effective, excellent treatment alternatives in patients whose tumors display a selected mutational profile [2].

Pediatric tumors, especially pediatric solid tumors have a lower mutational rate than adult tumors and uniform druggable mutations have not been identified yet, which until now precluded the use of targeted drugs in this patient population [3, 4]. However, mutations are obviously not the only molecular alterations that drive tumor development. In this regard, expression-based tumor profiling offers the advantage of deciphering deregulated pathways beyond genomics, including epigenetic and transcriptional regulation [5, 6]. Here we employed an array-based expression profiling approach to identify targetable aberrations in patients with refractory pediatric sarcomas. We assessed survival, efficacy as well as tolerability of treatment in patients who received targeted therapy in comparison to those who did not.

## RESULTS

### Patients

Twenty patients with refractory progressive pediatric sarcomas were eligible for targeted therapy (12 male and 8 female patients). The primary diagnosis was Ewing sarcoma (ES) in ten patients, osteosarcoma (OST) in five, and soft tissue sarcomas (STS) in another five, comprising three rhabdomyosarcoma (RMS), one synovial sarcoma (SYS), and one fibrosarcoma (FS) patient. Age at first diagnosis ranged between 6-26 years, while age at enrollment ranged between 8-35.5 years. The interval between first diagnosis and enrollment ranged between 1-15.5 years. All patients experienced at least their second relapse and/or were progressive and refractory to conventional treatment at the time of enrollment. Previous therapy included multiagent chemotherapy according to the EURO-E.W.I.N.G.99, Ewing 2008 protocols in eight ES patients with three having undergone total body MRI-governed involved compartment irradiation combined with high-dose chemotherapy and stem cell rescue (Meta-EICESS). One ES patient had received the AEWS0031 protocol, and one received parts of the Ewing 2008 protocol. Five OST patients had received previous chemotherapy according to EURAMOS-1/COSS-86 protocols with one patient additionally receiving multiagent chemotherapy according to CWS protocols for relapse. Three rhabdomyosarcoma (RMS) patients and one synovial sarcoma (SYS) patient received multiagent chemotherapy according to the CWS high risk protocol. The fibrosarcoma (FS) patient received a vast variety of Italian chemotherapy protocols.

Local radiotherapy (photon and/or proton therapy) of the primary tumor and/or the metastases had been performed in 14/20 patients. All patients except one ES patient underwent previous surgery to resect primary tumor and/or metastases. Three patients had received previous local hyperthermia treatment. A full summary of patient characteristics and previous therapies is provided in Supplementary Table 1.

Nine patients received targeted therapy (TT) based on gene expression analysis of tumor tissues while in eleven patients no targeted therapy was administered (non TT). Reasons for precluding patients from TT are detailed as follows: Severe sepsis developed in PT11 precluding other therapy potentially interfering with the immune response. Targeted therapy was not administered due to patient preference in patients 11, 13, 14, 18, 19 and 20. PT15 suffered from difficulty swallowing and was not able to receive oral therapy and received palliative care according to standard as described. In PT16 targeted therapy was not given due to family’s choice, instead the patient received 17 doses of Mifamurtid (2mg/m^2^) and best palliative care. Due to his HLA-type PT17 was eligible for an immunotherapy protocol with tumor specific T-cells and preferred this therapy. In PT12 chemotherapy with two cycles of trabectidin/irinotecan [7] was preferred over targeted therapy (physician’s choice). Survival after start of this therapy was four months. Target analysis could not be performed on the first biopsy of PT10 due to RNA degradation and by the time material from a repeated biopsy revealed targets, the patient had passed away. Targeted therapy was given as compassionate use in all patients.

Patients receiving targeted therapy did not differ from those who did not receive targeted therapy in the following risk factors: diagnosis, age, interval between first diagnosis and enrollment, disease state at enrollment and number of relapses (Table 1). The analysis of relapse risk with regard to the type of underlying sarcoma revealed the following: for the most frequent sarcoma (ES), which also has the shortest life expectancy amongst the three refractory sarcomas addressed here, the disease state was exactly identical in the TT group and the non TT group, i.e. the mean number of relapses was 2,4 in both groups. 5/9 sarcomas were ES in the TT group compared to 5/11 in the non TT group. In the TT group were two patients whereas in the non TT group was one patient with refractory progressive primary ES. In addition, overall expression analysis did not reveal any significant difference between TT and non TT patients (Supplementary Figure 1). Moreover, TOP2A expression was significantly higher in the TT group compared to the non TT group (Supplementary Figure 2). In a comprehensive genome-wide analysis TOP2A expression was found to be most predictive for a poor outcome [8]. Thus overexpression of TOP2A seems disadvantageous for the TT patients.

**Table 1:** Risk factors and baseline characteristics in patients who received targeted therapy (TT) and those who did not (non TT)

Risk factor	TT	non TT	P value
gender	5 male vs. 4 female	7 male vs. 4 female	0.71^*^
age at first diagnosis in years (mean ± SD)	17.2 ± 7.3	13.8 ± 3.9	0.2^†^
age at enrollment in years (mean ± SD)	21.1 ± 6.7	18.6 ± 6.6	0.42^†^
interval between first diagnosis and enrollment in years (mean ± SD)	3.6 ± 2.9	4.8 ± 4.8	0.48^†^
disease state at enrollment (number of relapses)	2.4	3.2	0.22^†^
sarcoma subgroup (ES compared to other entities as a risk factor)	5 ES vs. 4 non-ES (2 OS and 2 STS)	5 ES vs. 6 non-ES (3 OS and 3 STS)	0,65^*^
TOP2A Expression (mean ± SD)	326 ± 132	119 ± 79	0,004^†^

A Chi square test was used to compare the gender distribution. A two-tailed Student’s t-test was used to compare age at first diagnosis, age at enrollment, interval between first diagnosis and enrollment and disease state at enrollment. TOP2A is associated with poor outcome.

^*^P value of Chi Square test.

^†^P value of a two-tailed *t*-test.

### Targets

Target analysis was mostly based on newly obtained biopsies from the recent progression/metastasis. Addressable targets were identified in all analyzed biopsies.

**Figure 1:**
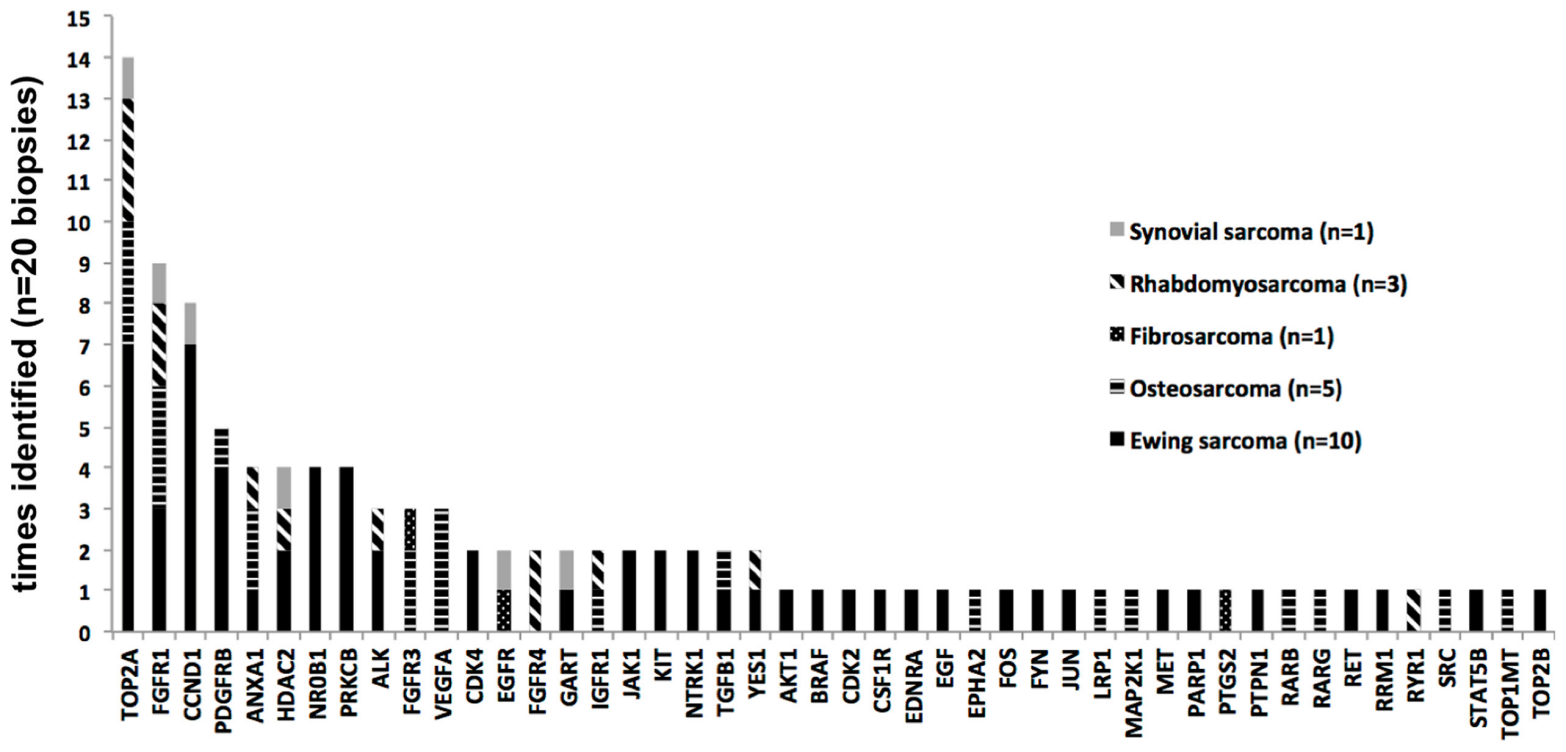
Most commonly identified targets and their distribution across biopsies

TOP2A (DNA topoisomerase II alpha) and FGFR1 (fibroblast growth factor receptor 1) were the most frequently upregulated targets across all analyzed sarcoma samples (n=14/20 and n=9/20 biopsies, respectively). The upregulation as well as the presence of an oncogenic mutation of the latter has also been detected as a feature of Ewing sarcoma in our previous studies [9]. CCND1 (cyclin D1) and PDGFRB (platelet derived growth factor receptor beta) were also upregulated mostly in ES biopsies (n=8/20, n=5/20), while VEGFA (vascular endothelial growth factor A) was mostly upregulated in OST biopsies (n=3/20). Other targets included ANXA1 (annexin A1), HDAC2 (histone deacetylase 2), ALK (anaplastic lymphoma receptor tyrosine kinase), FGFR3 (fibroblast growth factor receptor 3), NR0B1 (nuclear receptor subfamily 0 group B member 1) and PRKCB (protein kinase C beta). A summary of identified targets with respect to tumor type is provided in Figure 1.

### Prescribed medications

**Figure 2:**
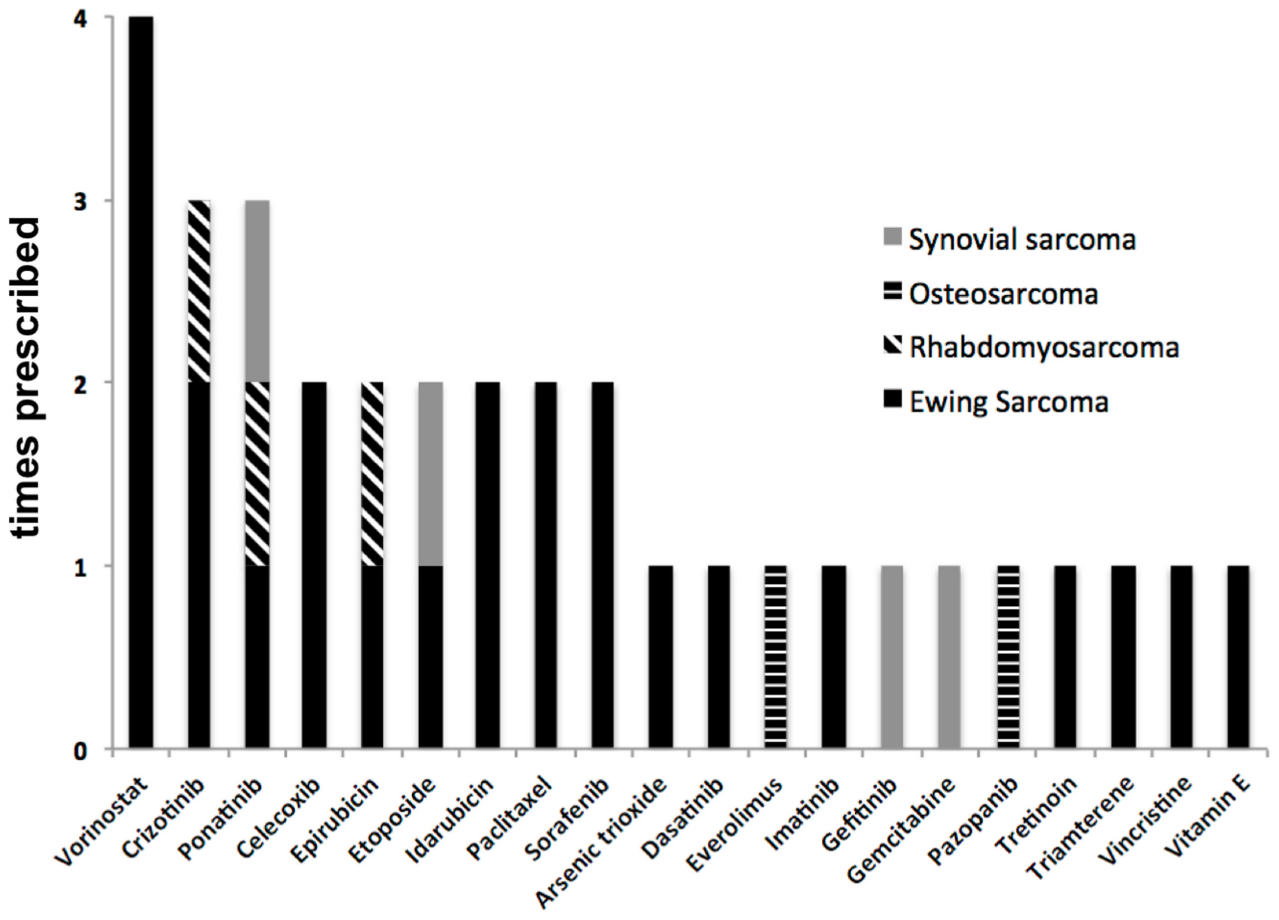
Prescribed drugs according to diagnosis group (n=9 treated patients)

Twenty different agents of various drug classes were administered in the patients treated based on target therapy recommendation. Most of these agents were kinase inhibitors including tyrosine kinase inhibitors (Ponatinib, Dasatinib, Gefitinib and Pazopanib, Crizotinib, Sorafenib and Imatinib). Cytotoxic agents including topoisomerase II inhibitors (Epirubicin, Etoposide and Idarubicin) were also frequently prescribed. The selection of agents of the same substance group (e.g. epirubicin vs idarubicin) was based on previous use in the patients as described in the methods section (selection of therapeutics), i.e. epirubicin was selected in case of previous use of idarubicin. Other cancer drugs included the mTOR inhibitor Everolimus, the taxane Paclitaxel and differentiation inducers including a histone deacetylase inhibitor (Vorinostat) and an all-trans retinoic acid (Tretinoin). Furthermore, a nucleoside analog (Gemcitabine), a nonsteroidal anti-inflammatory drug (Celecoxib), a diuretic (Triamterene), a metalloid oxide (Arsenic trioxide) and a vitamin (Vitamin E) were used [10]. Prescribed drugs are summarized in Figure 2. Table 2 summarizes the identified targets in each of the treated patients and the drug applied for each target.

**Table 2:** Summary of the identified targets in each of the treated patients and the corresponding applied drug and its dosage

Patient	Diagnosis	Target	Drug	Dosage	Comment
PT1_1	Ewing sarcoma	HDAC2	Vorinostat	300 mg p.o.	reduced dosage
		STEAP1	Paclitaxel	200 mg/m^2^ i.v	standard dosage
PT1_2	Ewing sarcoma	HDAC2	Vorinostat	400 mg p.o.	standard dosage
		BRAF	Sorafenib	400 mg p.o.	reduced dosage
		NR0B1	Tretinoin	25 mg/m^2^ p.o.	reduced dosage
		SCNN1G	Triamterene	25 mg p.o.	reduced dosage
PT1_3	Ewing sarcoma	CCND1	Arsenic trioxide	0,1 mg/kg p.o.	reduced dosage
		PRKCB	Vitamin E	400 IE p.o.	standard dosage
PT1_4	Ewing sarcoma	ALK	Crizotinib	500 mg p.o.	standard dosage
PT2	Ewing Sarcoma	HDAC2	Vorinostat	100 mg p.o.	reduced dosage
		FGFR1	Ponatinib	15 mg p.o.	reduced dosage
		TOP2A	Idarubicin	5,5 mg/m^2^ p.o.	reduced dosage
		STEAP1	Paclitaxel	175 mg/^2^m i.v.	reduced dosage
PT3	Ewing sarcoma	ALK	Crizotinib	400 mg p.o.	reduced dosage
		TOP2A	Etoposide	2x25 mg/m^2^	standard dosage
		FGFR1	Sorafenib	400 mg p.o.	reduced dosage
PT4	Rhabdomyosarcoma	MET	Crizotinib	500 mg p.o.	standard dosage
		TOP2A	Epirubicin	100 mg/m^2^	standard dosage
		FGFR1	Ponatinib	45 mg p.o.	standard dosage
PT5	Synovial sarcoma	FGFR1	Ponatinib	30 mg p.o	reduced dosage
		RRM1	Gemcitabine	800 mg/m^2^ i.v.	reduced dosage
		TOP2A	Etoposide	2x25 mg/m^2^ p.o.	standard dosage
		EGFR	Gefitinib	250 mg p.o.	standard dosage
PT6	Ewing sarcoma	TOP2A	Idarubicin	n.d.	n.d.
		STEAP1	Paclitaxel	n.d.	n.d.
PT7	Ewing sarcoma	RET	Imatinib	400 mg p.o.	standard dosage
		PDPK1	Celecoxib	200 mg p.o.	standard dosage
		PDGFRB	Dasatinib	100 mg p.o.	standard dosage
		TOP2A	Epirubicin	100 mg/m^2^ i.v.	standard dosage
PT8	Osteosarcoma	SRC, MAP2K1	Everolimus	5 mg p.o.	reduced dosage
		Sorafenib	PTEN	400 mg p.o.	reduced dosage
PT9	Osteosarcoma	Pazopanib	FGFR3	400 mg p.o.	reduced dosage
		Palbociclib	Cdkn2a/b	100 mg p.o.	reduced dosage

Standard dosage: according to manufacturers recommendation for approved indication. Reduced dosage: reduction according to toxicity, drug interactions and age.

### Therapies, adverse events, quality of life, and response

PT1 with ES, due to three biopsies during the course of his disease, received 4 different targeted therapy-regimens and will therefore be described in more detail. PT1 first received Vorinostat and Paclitaxel given as a combination with the chemotherapeutic Vincristine as based on target pathway analysis of his biopsy. This therapy led to stable disease according to RECIST 1.1 criteria for seven months. Due to disease progression in later follow up imaging studies a rebiopsy was performed and a new targeted therapy with Vorinostat, Sorafenib, Triamterene and Tretinoin was started also resulting in a stable disease that lasted for another two months. As radiologic progression was observed, targeted therapy combination with Arsenic trioxide and Vitamin E based on the same biopsy analysis was recommended. However, therapy was delayed due to a pneumonia that required intravenous antibiotic therapy. Shortly after therapy start, disease progression was observed and a new chemotherapy combination was attempted under which disease progression continued. Again based on the same biopsy analysis and due to a high ALK expression, therapy with Crizotinib was initiated. However, the patient suffered from increasing bone pain and episodes of dyspnea and was referred to palliative care with discontinuation of the Crizotinib therapy, due to physicians preference. The grade 4 (G4) bone pain, grade 3 (G3) pneumonia with dyspnea were classified as unlikely related to the targeted therapy. Other G3 AEs included lymphopenia, thrombocytopenia classified as certainly related to targeted therapy and activated partial thromboplastin time prolongation classified as possibly related to targeted therapy. The patient also suffered from grade 2 (G2) anemia classified as possibly related to targeted therapy.

PT2 with ES received a combination therapy of Vorinostat, Ponatinib, Idarubicin and Paclitaxel but demonstrated progressive disease at the primary tumor site upon radiologic evaluation. AEs included G3 lymphopenia and G2 neutropenia classified as certainly related to targeted therapy.

PT3 with ES received a combination of Crizotinib, Etoposide and Sorafenib and also continued to have progressive disease. Sorafenib was preferred for FGFR1 blockade due to side effect profile and pharmacological interactions. AEs due to targeted therapy were not observed.

PT4 with RMS received Crizotinib, Epirubicin and Ponatinib under which he experienced stable disease for almost seven months in follow up imaging studies. AEs included G4 neutropenia and G2 lymphopenia classified as certainly related to targeted therapy as well as G3 elevation of AST levels and G2 elevation of bilirubin classified as probably related to targeted therapy.

PT5 with SYS received Gemcitabine, Etoposide, Ponatinib and Gefitinib and continued to experience progressive disease in follow up imaging. His AEs included G4 neutropenia and G3 anemia, lymphopenia and thrombocytopenia all classified as certainly related to targeted therapy. Additionally, a G2 cough and a pericardial effusion as well as a grade 1 (G1) pleural effusion were observed and classified as unlikely related to targeted therapy.

PT6 with ES received a combination of Idarubicin and Vorinostat, however returned to home country precluded a sufficient and accurate follow up evaluation of side effects and QOL analysis according to investigator standards. OS and PFS information was available.

PT7 with ES received Imatinib, Dasatinib, Epirubicin and Celecoxib. However, due to G4 psychological AEs of anxiety and depression that were classified as certainly related to Imatinib therapy, Imatinib therapy was discontinued and later on resumed with a 50% dose reduction. G4 hematologic AEs such as anemia, neutropenia, lymphopenia and thrombocytopenia classified as certainly related to therapy resulted in discontinuation of Dasatinib. Radiotherapy of the shoulder was started in order to palliate local symptoms due to metastasis leading to discontinuation of Epirubicin therapy after one dose. Therapy with all four medications resumed in its full dose after the end of radiotherapy. Additional AEs included G2 vomiting and nausea classified as certainly related to therapy. Progressive disease continued upon radiologic evaluation.

Therapy in PT8 and PT9 was administered based on RNA overexpression of the therapeutic target despite the identification of genetic alterations on DNA level (deletion mutations in CDKN2A/B and PTEN). On initial follow up PT8 experienced stable disease for seven months under therapy with Everolimus, which later on developed progressive disease. PT8 experienced G2 pancytopenia including G3 thrombopenia and G4 neutropenia as well as not classified bone pain and cardiac insufficiency all certainly related to therapy. Moreover, PT8 suffered from G2 hand-food-syndrome and G2 neuropathic pain probably related to targeted therapy.

PT9 experienced mixed response under therapy with Pazopanib resulting in stable disease of lung metastasis for five months while disease continued to progress in the abdominal wall despite the fact that the biopsy for the analysis was obtained from the abdominal wall. PT9 suffered from inappetence, fatique, nausea and neutropenia which were certainly related to therapy.

AEs were not adequately documented in patient 3, 6, 14, 18 and 19. All AEs and their relation to targeted therapy are summarized in Supplementary Table 2.

Patients who did not receive targeted therapy went to palliative therapy according to local standard of care. Patients were predominantly cared for on an outpatient basis or visited at home by the hospitals' palliative care team consisting of a palliative care physician, palliative care nurse, and social workers. Primary goal was to maintain QOL as long as possible. Therefore, pain management (including opiates and tetrahydrocannabinol) and symptom control were the major focus. Medication was adapted to the individual patients needs. Psychosocialcare was offered and implemented based on the patients demand. These patients suffered from disease related AEs which are summarized in Supplementary Table 2.

**Figure 3:**
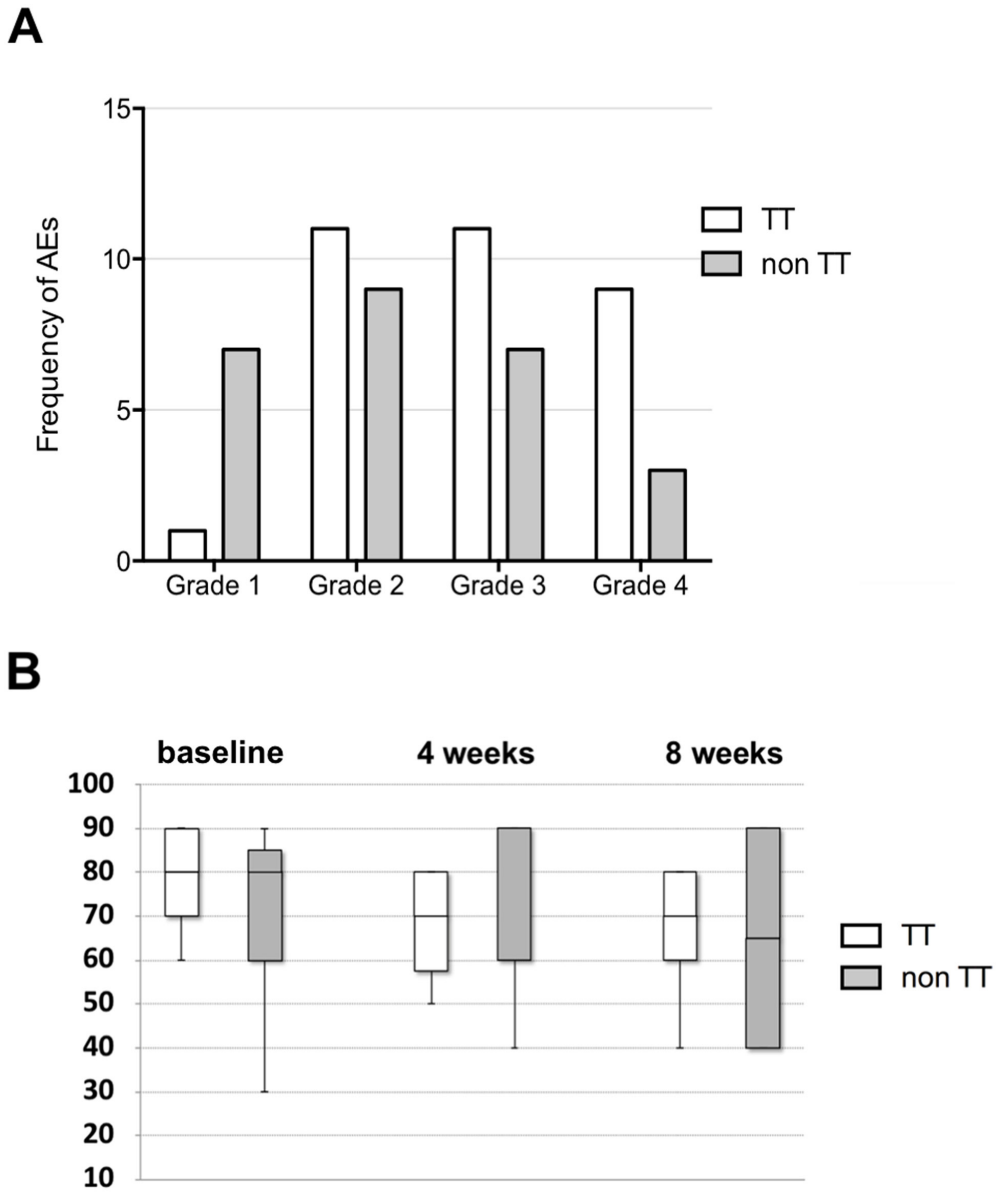
Adverse events and quality of life **(A)** Frequency of adverse events in patients receiving targeted therapy (TT) compared to those who did not (non TT) (total number of n=15 evaluable patients). The frequency of Grade 2, 3 and 4 AEs did not differ significantly between the two patient groups (Chi square P=0.98, 0.54 and 0.12 respectively). However, Grade 1 AEs were significantly less in patients receiving targeted therapy (Chi square P=0.009). **(B)** QOL is assessed by comparing performance status according to Karnofsky/Lansky status between patients receiving targeted therapy (TT) and those who did not (non TT). Performance status was not significantly different at baseline (P=0.33) as well as at four (P=0.96) and eight weeks (P=0.89) following enrollment/ start of therapy between the two patient groups.

The frequency of G2, G3 and G4 AEs did not differ significantly between the two patient groups (Chi square P=0.98, 0.54 and 0.12 respectively). However, G1 AEs were significantly less in patients receiving targeted therapy (Chi square P=0.009). Figure 3A compares the frequency of AEs between the two patient groups.

QOL was assessed by performance status according to Karnofsky/Lansky. This did not differ significantly at baseline as well as at four and eight weeks following enrollment/start of therapy between the two patient groups (P=0.33, 0.96 and 0.89 respectively). Figure 3B shows the performance status of the two respective patient groups.

### Survival

**Figure 4:**
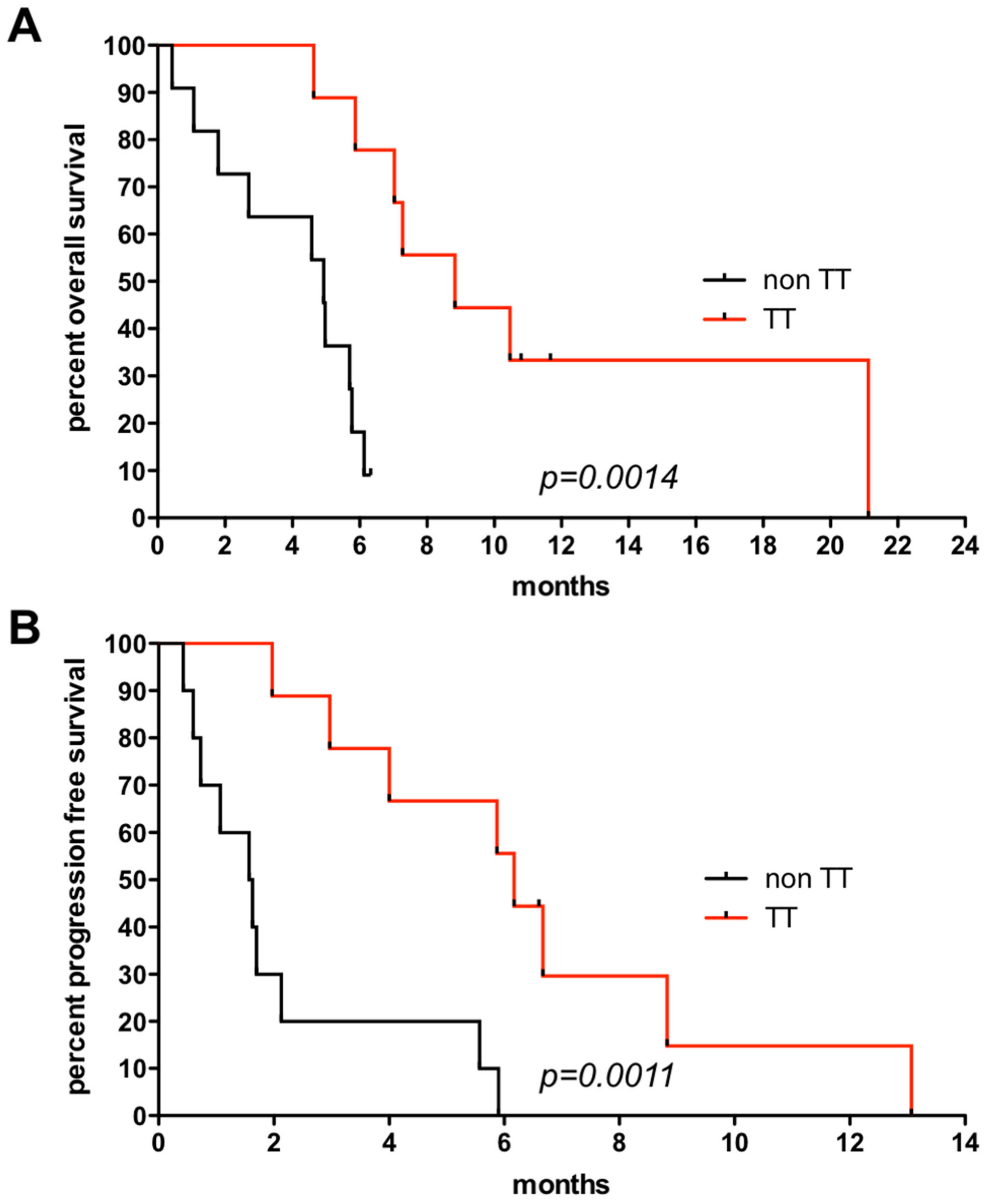
Survival analysis **(A)** Overall survival (OS) using the Kaplan-Meier estimator between patients receiving targeted therapy (TT=9) and those who did not (non TT=11). Median OS=8.83 months in the TT group versus 4.93 months in the non TT group. Log-rank P=0.0014, Gehan-Breslow-Wilcoxon P=0.0025, with a hazard ratio (HR) of 0.1435 and a 95% confidence interval (CI) ranging from 0.044 to 0.471. **(B)** Progression free survival (PFS) using the Kaplan-Meier estimator between patients receiving targeted therapy (TT=9) and those who did not (non TT=10 patients since PFS was not available for PT18). Median PFS=6.17 months in the TT group versus 1.6 months non TT group. Log-rank test (P=0.0011) and the Gehan-Breslow-Wilcoxon test (P=0.0015) with a HR of 0.1448, 95% CI of 0.045 to 0.463.

OS was significantly longer in patients receiving targeted therapy as evaluated both by a log-rank (Mantel-Haenszel/ Mantel-Cox) (P=0.0014) and the Gehan-Breslow-Wilcoxon test (P=0.0025) with a hazard ratio (HR) of 0.1435 and a 95% confidence interval (CI) ranging from 0.044 to 0.471. Median OS was 8.83 months in the TT group (n=9 patients) versus 4.93 months in the non TT group (n=11 patients), respectively. Median OS in our TT group also exceeded survival of historical control groups with different therapy regimens [7]. PFS was also significantly different between the two groups with a log-rank test (P=0.0011) and the Gehan-Breslow-Wilcoxon test (P=0.0015) with a HR of 0.1448, 95% CI = 0.045 to 0.463. Median PFS was 6.17 months and 1.6 months in the TT group (n=9 patients) versus non TT group (n=10 patients since PFS was not available for PT 18), respectively. Kaplan-Meier estimates are given in Figure 4 for OS (Figure 4A) and PFS (Figure 4B).

## DISCUSSION

Sarcomas account for around 13% of malignancies in children, adolescents and young adults compared to 1% of all malignant tumors including adults [11]. Despite its high prevalence in children and adolescents, in general very little improvement has been made in the past 25 years with regards to survival of patients with bone and soft tissue sarcomas. The 5-year survival rate of osteosarcoma in children and adolescents has stagnated at around 67% since 1990. A similar pattern is observed for ES in adolescents with 5-year survival around 59% since 1995. An increase from 47% to 74% in 5-year survival is observed for children (under 18 years of age) with ES between 1990-1994 with stable rates thereafter. For RMS, survival rate in children has been steady around 68% since 1982 while stagnating around 48% in adolescents since 1995 [12].

With the exception of Kaposi sarcoma, adolescents and young adults (18 to 39 years of age) soft tissue sarcomas appear to have benefitted the least from recent treatment advances with the average annual percent change in 5-year survival declining for age groups between 15-45 years while increasing in older and younger groups. The age-dependent survival improvements have been directly correlated with clinical-trial accrual patterns suggesting a link to limited participation of this age group in clinical trials. The decrease in survival improvement for all cancer types is most prominent in patients between 25-35 years [13, 14]. These statistics are consistent between the US and Europe [15, 16].

Improvement in outcomes during the last 50 years seems to be most prominent in patients with localized pediatric sarcomas. Prognosis remains very poor for patients with metastatic or relapsed disease [17, 18].

Complex multimodal therapy regimens have contributed to very modest improvement in cancer survival rates. This improvement however, has been accompanied by mutilation and severe late effects of cytotoxic therapies, in particular after radiotherapy. Thus, we need a deeper understanding of the biological abnormalities underlying the individual disease to specifically address those abnormalities on a molecular level. The development and implementation of targeted therapies may help to overcome both toxicity and resistance.

Several classes of targeted therapies showed considerable efficacy against multiple cancer types in large clinical trials and soon afterwards many have gained FDA approval marking a new era of precision cancer medicine [19]. Those targeted therapies comprise drugs that are immunologically active and differentiation-inducing, as well as drugs that block angiogenesis. Others inhibit single-strand break repair and block DNA replication through interaction with the poly ADP ribose polymerase protein (PARP) or a certain signal transduction pathway.

With the completion of the cancer genome project it became clear that the biological processes underlying several cancer types are based on common hallmarks [20, 21]. The discoveries made through that project enabled the broad application of many targeted cancer therapies to different types of malignancies that harbor the same genetic abnormality. This led to a shift in the conventional clinical trial design into more complex molecularly based strategies that explore treatment options for multiple cancer types with the same genetic aberration coined “basket trials”. These allow the study of treatment options in rare cancer types difficult to evaluate in randomized controlled trials. Another novel design is the so-called “umbrella trial”, wherein such trials focus on a single entity but test for several treatment options based on the presence of predefined molecular profiles [22, 23].

With today’s array and sequencing technologies, it is possible to rapidly and comprehensively screen tumor tissues for many genetic aberrations and expressional changes at decreasing costs enabling a more precise biological view of each patient’s tumor. These technologies have led to the identification of molecular disease subclasses not previously recognized [24].

With the vast library of existing targeted therapies, it is highly important to choose the most suitable drug for each patient’s tumor, thus increasing efficacy and reducing undesired adverse events. Indeed, a number of trials with innovative design have been recently undertaken employing molecular tumor profiling to guide therapeutic decisions in patients with relapsed or refractory tumors [25, 26], ClinicalTrials.gov Identifier: NCT02091141. Some of those trials have been expanded and others are currently underway to specifically target the pediatric patient population [27], ClinicalTrials.gov Identifier: NCT02813135.

It is noteworthy that of the 100 patients with refractory pediatric cancer enrolled in the multicenter individualized cancer therapy (icat) study, potential actionable alterations were found only in 31% with copy number alterations and deleterious mutations most commonly identified [28]. We found that actionable genomic alterations may be as low as 10% or less in pediatric sarcoma (Groebner et al., Nature in press).

Conversely, target identification was possible in all 20 patients of our single center study due to the use of gene expression analysis and the employment of novel pathway and target identification software [29].

The microarray technology used for the gene chips has high technical reproducibility and produces robust gene expression data thus enabling the comparison of multiple samples [30].

Our results suggest that patients receiving targeted therapies based on expression analysis of their tumors may have a significantly higher OS and PFS than those who did not. Although special care was taken to specifically rule out that patients, who did not receive targeted therapy had more advanced disease including disease entities, number of relapses and number of patient with refractory progressive primary disease, we cannot completely rule out that risk factors hitherto not considered or unknown convey a bias into the analysis, warranting a randomized study. The only risk factor that we found to be distributed significantly different in both groups was a disadvantage for the TT patients: the TOP2A expression. A higher TOP2A expression as seen in the TT group is a strong predictor for a poor outcome [8]. Despite significant prolongation in OS and PFS in patients receiving array recommended targeted therapies the disease continued to progress in most patients, while stable disease was only achieved in three patients (33%) for at least seven months. However, this might be partly due to the advanced disease state in most patients. Indeed, the personalized targeted therapy was only given as compassionate use in highly refractory heavily pretreated patients.

Within the small group adverse events were not significantly different in both groups. The QOL of those patients as assessed by Karnofsky/Lansky score was not affected by the targeted therapy making it a feasible therapy option in refractory cases.

The use of cytotoxic versus targeted agents in a refractory setting is a matter of debate. A meta-analysis of 143 phase I trials in children with refractory solid tumors compared the use of cytotoxic versus targeted drugs. Despite having a similar pooled estimate of stable disease rate and PFS, cytotoxic drugs demonstrated a 2.1-fold higher objective response rate and a 1.8-fold higher pooled estimate of dose-limiting toxicity rate (mostly G3-G4 hematologic toxicities) [31]. However, the majority of targeted drug trials in this analysis did not match the drugs to the molecular aberrations of interest in each patient’s cancer. It has been shown that matching targeted therapy to a documented molecular aberration in each patient results in a significantly higher response rate and longer PFS in adults with advanced cancer when compared to unmatched therapy choices [32, 33].

In this study, a minimum of three drugs was given as a combination for each patient in an attempt to avoid cancer escape through alternative pathways. The need for triple therapy has been suggested using mathematical models especially in patients with large disease burden [34]. Our current knowledge suggests that blocking a single target or pathway causes unwanted selection pressure on tumor cells and is unlikely to result in sustained responses especially in refractory cases [35]. A more dynamic and adaptive model of cancer therapy has been suggested in which a combination of two therapies is given sequentially at defined time points and as resistant subpopulations emerge during the treatment course [36]. Additionally, the analysis of a newly obtained biopsy is also key to successful target identification and a more effective treatment in our experience.

While previous trials provided variable efficacy data of molecularly guided therapy using mutation analysis and copy number alterations in patients with advanced cancer [37], our study provides evidence supporting the possible efficacy of transcriptome-based targeted therapy in patients with refractory pediatric sarcoma, which warrants further investigation of this methodology in a larger population of pediatric patients.

In summary, expression-based personalized targeted therapy provides a feasible option for patients with refractory pediatric sarcomas. When applied properly this therapy might provide a survival benefit without causing an increase in adverse events or a reduction in QOL. The investigation of the efficacy of this method is warranted in a larger patient population.

## MATERIALS AND METHODS

### Patients

Children, adolescents and young adults with refractory relapsed (two relapses or more) or progressive (i.e. resistant) primary pediatric sarcomas were assessed for their eligibility for expression based personalized targeted therapy through the internal and/or interdisciplinary tumor. The tumor board is comprised of pediatric oncologists, radiation oncologist, pathologists, surgeons, and respective organ specialists if needed. Informed consent for treatment in the IRB approved registry study (2562/09) was obtained from eligible patients and/or their primary care provider.

### Samples

Samples were obtained after informed consent from patients and/or their primary care provider. The local institutional review board approved sample collection and analysis. Tumor material was obtained from an index tumor site using minimal invasive biopsy or surgery when medically indicated. Snap frozen biopsy material was assessed by a soft tissue pathologist (KS) to confirm relapse/recurrence of the underlying sarcoma. Samples were further assessed for quality and tumor cell content and only tumor material with a tumor content higher than 70 % was subjected to microarray analysis. Only one sample per patient biopsy was analyzed. Target analysis was based mostly on newly obtained biopsies from a recent progression/metastasis.

### Gene expression analysis and target identification

Tumor RNA was extracted and hybridized using Affymetrix Human Gene ST1.0 arrays for 18 patients and using Affymetrix GeneChip U133 Plus 2.0 array in two patients. The array data was background adjusted, and normalized using Robust Multiarray Average (RMA) [38]. A fold change of gene expression was generated by comparing the expression values to the mean expression across 21 (GEO: GSE45544, Human Gene ST1.0 arrays) or 22 normal human tissues (GSE18674, U133 Plus 2.0 arrays), respectively. Machine learning-based software (TARGETgene) was applied to analyze the data and rank preferentially expressed genes according to their significance [29]. Online accessible tools such as KEGG and REACTOME allowed the visualization of different pathways and interactions between their components [39, 40]. A gene was identified as overexpressed if its expression was more than 2-fold overexpressed of the RMA normalized, linearized expression values. A target had to be identified as an overexpressed key gene in a cancer pathway (either within the first 100 ranked hub genes using TARGETgene and/or to have a known annotated function as a key gene in known cancer pathways using KEGG or REACTOME and/or is cited in the literature to play key role in cancer cell survival). When using TARGETgene, gene ranking was taken into account in ES samples as reference entities were incorporated into the software, whereas a high fold change was the key factor in OS and STS. Gene set enrichment analysis (GSEA) was performed on the data to assess possible involvement in key pathways that might infer therapy resistance or preferential response to certain therapeutic agents [41].

For comparison of overall expression data between the TT and non TT group the following approach was used: The heatmap of genes (Supplementary Figure 1) was generated using R (version 3.4.1; https://www.R-project.org/). The input data were annotated expression values, RMA normalized compared to normal tissue expression. The expression data of two samples (PT8, PT9) had to be merged to the expression data of the remaining samples as different platforms had been used (see above). Therefore, the data was filtered to remove probe sets without annotated gene symbol, and average expression was used for probe sets with non-unique gene symbols, respectively. Finally, the expression data of PT8 and PT9 was merged to the remaining samples based on gene symbols (17786 genes available). For further analysis, the log2 expression values were used and the whole dataset was quantile normalized (R package preprocessCore).

The dendrogram of samples was generated based on the expression levels of all 17786 genes using unsupervised, hierarchical clustering (euclidian distance, complete linkage).

The analysis for differential expression between the TT and the non TT group was conducted in R using the moderated t-statistics of the package limma (default settings). All 17786 genes were tested. To correct for multiple testing, the Benjamini-Hochberg method [42] was used. For the heatmap, the 50 top genes with smallest adjusted P values were selected. The plot depicting the Z-scaled expression of these 50 genes and the dendrogram of samples based on all genes was generated using the R package gplots.

### Selection of therapeutics

After target identification, targeted therapies for a certain target were matched using the Drugbank database and then weighed for efficacy through screening of the literature [43]. In order to provide the best evidence-based medication, criteria for therapy recommendation applied by the decision board included: availability on the market, drug delivery, no previous use in the patient, citations related to disease, citations related to other cancers, side effects, drug interactions, cumulative toxicity, oral application to allow best possible QOL and approval by the German authorities. A list of prioritized targets and their respective medications was generated prior to the presentation of the patient in the decision board. The decision board is comprised of three pediatric oncologists, two of whom are physician scientists (MD, PhD), one clinical pharmacologist, a scientist (PhD in biochemistry/Immunology), and a doctoral student to collect the data. Drug combinations were based on pharmacologic expertise to avoid undesired drug interactions, and adverse events with special attention paid to cumulative dose of previous treatments. Order and timing of the respective drugs was organized accordingly. Potential sensitization of tumor by one drug for increased effect of another drug was taken into consideration. Therapy was given as compassionate use in highly refractory patients.

### Therapy and follow up

Follow up measures were implemented within the in-patient as well as during out-patient visits and a detailed plan for monitoring of possible side effects was established and individualized for each patient. Therapy compliance and disease progress were closely examined for most patients, both clinically and through imaging studies according to the criteria of best clinical practice. Response evaluation was performed using the Response Evaluation Criteria in Solid Tumors RECIST 1.1 at four week intervals [44]. Follow up was performed at four-week intervals. Patients who did not receive targeted therapy were also followed up to assess their survival and QOL.

### Quality of life and adverse events

Adverse events (AEs), their severity and their attribution to therapy were assessed following targeted therapy as they occurred and at repeated four week intervals using the NCI-CTCAE v.4.03: June 14, 2010 [45]. An adverse event is defined as any untoward medical occurrence associated with the use of a pharmaceutical product and which does not necessarily have to have a causal relationship with it. The causal relationship was assessed according to the causality assessment system proposed by the World Health Organization Collaborating Centre for International Drug Monitoring, the Uppsala Monitoring Centre (WHO–UMC) as certain, probable, possible, unlikely, conditional and unassessable [46]. The frequency of adverse events at the predefined time point was compared using a Chi-square test. Performance status was evaluated according to the Karnofsky (age ≥ 16 years) or the Lansky scale (age < 16 years) in all enrolled patients at the time of enrollment/therapy begin and in four week intervals [47, 48]. For the comparison of performance status at baseline as well as performance change, a two-tailed t-test to compare means assuming equal variances was used.

### Survival analysis

Overall survival (OS) was defined as the time from enrollment to the time of death or last follow up and was compared using the Kaplan-Meier estimator in two similar patient groups: patients who received targeted therapies and patients who did not.

Progression free survival (PFS) was defined as the time from enrollment until radiological confirmed disease progression or death and was compared between the two groups using the Kaplan-Meier estimator. Survival analysis was performed and plotted using GraphPad prism software V6.0h.

Special care was taken to compare all known relevant patient and prognostic factors between both groups (Table 1).

### Data access

Microarray data used in this study is deposited in GEO (GSE45544, GSE73166).

## SUPPLEMENTARY MATERIALS FIGURES AND TABLES






