# Supplementary material for: Transcriptome based individualized therapy of refractory pediatric sarcomas: feasibility, tolerability and efficacy
Source: Oncotarget. 2018 Apr 17;9(29):20747–60. doi: 10.18632/oncotarget.25087 (PMC5945512; doi:10.18632/oncotarget.25087)
Supplement: Supplementary file 1 [file oncotarget-09-20747-s001.pdf]

Transcriptome based individualized therapy of refractory pediatric sarcomas: feasibility, tolerability and efficacy

SUPPLEMENTARY MATERIALS

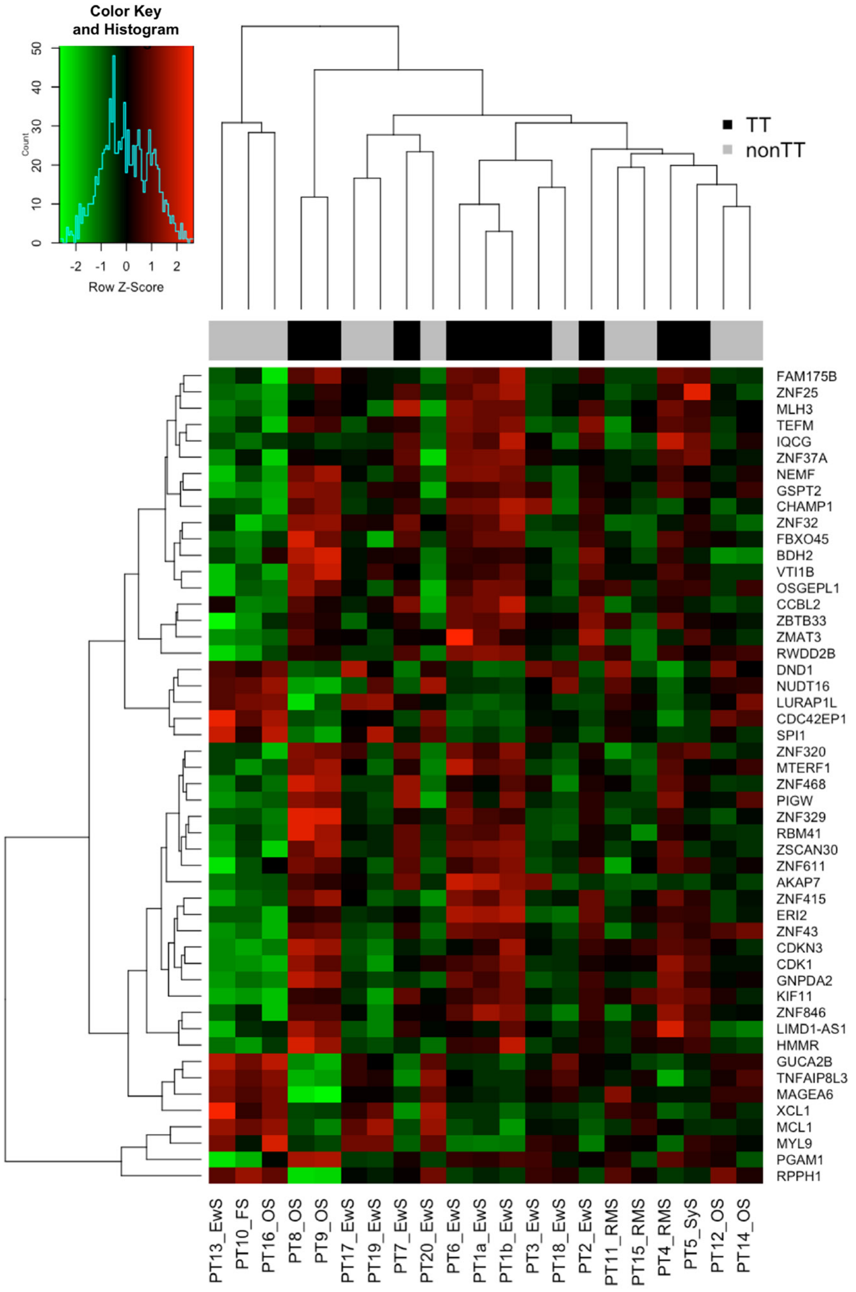

Supplementary Figure 1: This heatmap shows the expression levels of a group of genes in all 21 samples (of 20 patients) adapted to their expression in normal tissue (see Materials and Methods). Here, the 50 top genes obtaining smallest adjusted P values in differential expression analysis between the TT group of patients and the non TT group are illustrated (overall smallest adjusted P value=0.059 for ZNF320). In the heatmap, red colors indicate a relatively high expression of a gene in one sample compared to the other samples, and green colors indicate a relatively low expression. The genes are clustered by expression levels, visualized by the dendrogram on the left. Above the heatmap, the dendrogram of samples is plotted. This unsupervised clustering of samples is based on the expression of all 17786 available genes. Additionally, the group assignments of the patients are indicated; patients belonging to the TT and non TT groups are marked in black and gray, respectively.

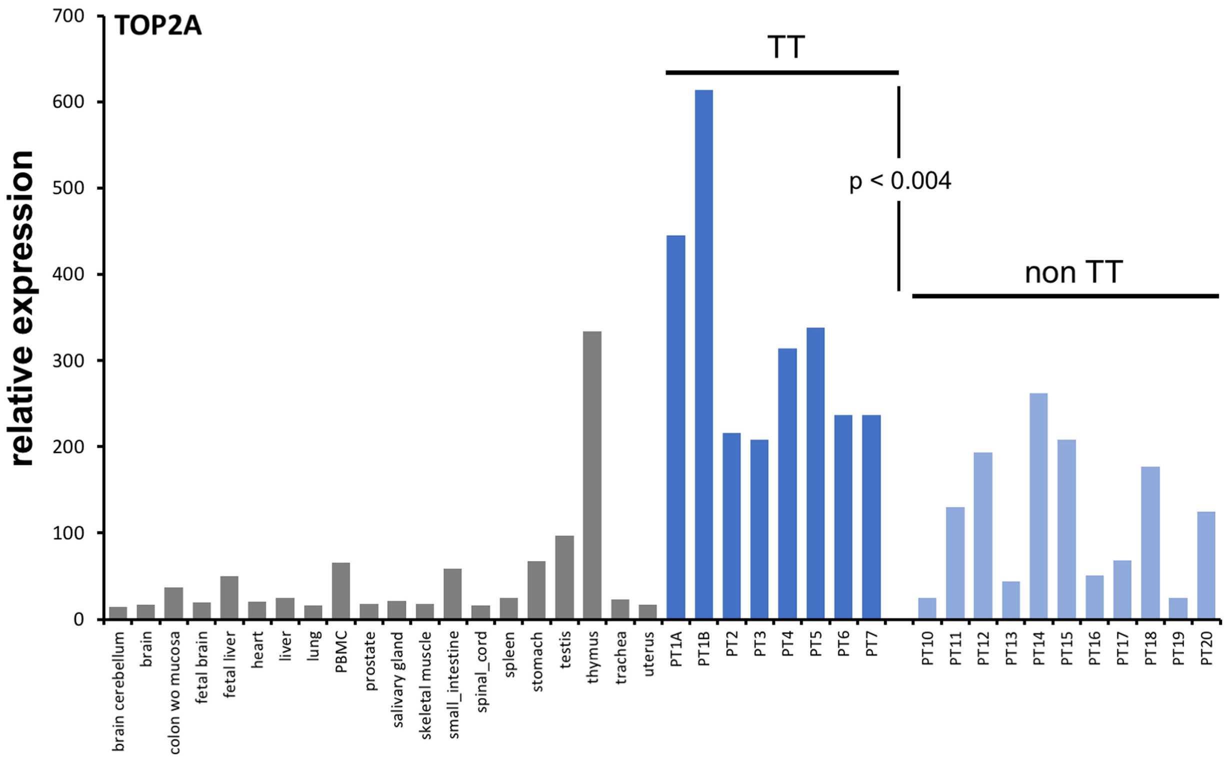

Supplementary Figure 2: *TOP2A* gene expression in treatment groups. Expression profile of TOP2A in TT (blue) and non TT (light blue) patient groups in comparison to normal tissue (gray). Patient RNA were hybridized onto Human Gene ST1.0 arrays (Affymetrix; GSE45544, GSE73166) and compared to a published microarray study of normal tissue (GSE45544). Significance of expression difference between TT and non TT groups was analyzed by *t-test.* Each bar represents the expression signal of an individual array.

Supplementary Table 1: Detailed patient characteristics. Patients PT1 to PT9 received targeted therapy, Patients PT10 to PT20 did not receive targeted therapy.

See Supplementary File 1

Supplementary Table 2: Adverse events and their attribution to therapy in evaluable patients.

See Supplementary File 2
